# Resequencing and Functional Analysis Revealed That *BsDFR4* Could Cause the Formation of Different Flower Colors in *Bletilla striata* (Orchidaceae)

**DOI:** 10.3390/ijms26083555

**Published:** 2025-04-10

**Authors:** Siting Zheng, Zeyuan Mi, Yuanqing Chang, Ruohan Huang, Jiaxin Li, Xiulin Jiang, Shuai Liu, Zhezhi Wang

**Affiliations:** National Engineering Laboratory for Resource Development of Endangered Crude Drugs in Northwest China, Key Laboratory of the Ministry of Education for Medicinal Resources and Natural Pharmaceutical Chemistry, College of Life Sciences, Shaanxi Normal University, Xi’an 710119, China; zhengsiting@snnu.edu.cn (S.Z.); mizeyuan@snnu.edu.cn (Z.M.); cyqingfei@163.com (Y.C.); huangruohan@snnu.edu.cn (R.H.); jiaxinli@snnu.edu.cn (J.L.); silllimjiang@163.com (X.J.)

**Keywords:** *Bletilla striata*, DFR, anthocyanins, flower color formation, resequencing

## Abstract

The formation of flower color is closely related to anthocyanin synthesis. In this study, flowers of *Bletilla striata* (Orchidaceae) exhibiting distinct color morphs were collected and analyzed. The HPLC results showed significantly higher total flavonoid and anthocyanin contents in purple flowers compared to pink counterparts, with increases of 2.20-fold (*p* < 0.01) and 15.22-fold (*p* < 0.01), respectively. Cyanidin was the predominant anthocyanin in *B. striata*. Resequencing analyses highlighted SNP as the primary variation associated with color divergence. A comprehensive screen identified 61 genes encoding enzymes critical to the flavonoid and anthocyanin biosynthesis pathways in *B. striata*. Among these, 16 flower-specific genes exhibited high expression levels and harbored SNP variations. Notably, a premature stop codon was identified in a gene encoding dihydroflavonol 4-reductase (DFR), leading to truncated protein synthesis and potential disruption of anthocyanin production. Further, the heterologous overexpression of *BsDFR4* in *Phalaenopsis aphrodite* changed petal color from white to yellow-green, demonstrating that it indeed played a regulatory role in the formation of flower color. Furthermore, yeast one-hybrid assays confirmed that transcription factors *BsMYB36* and *BsMYB51 could* directly bind to the *BsDFR4* promoter, suggesting their synergistic regulation of anthocyanin biosynthesis. These results provided a conceptual basis for insights into the formation of different flower colors in Orchidaceae.

## 1. Introduction

Flowers are unique reproductive organs of angiosperms and play an important role in plant growth and development. Especially for ornamental plants, color directly affects their ornamental and economic value [1]. The formation of flower color is closely related to the tissue structure of petals, the type, and the distribution mode of pigment in petal cells [1,2]. The pigments in nature can be mainly classified into three categories: flavonoids, carotenoids, and betalains [3,4,5,6], among which the former two are dominant in Orchidaceae [7]. Flavonoid is a kind of water-soluble natural pigments that produce a full spectrum of colors from pale yellow to blue-purple [1,8]. Anthocyanins are the most important flavonoid pigments synthesized in the cytoplasm and stored in the vesicles, which are the main substance affecting flower color, controlling the production of red, purple, and blue flower colors [1,6,9,10]. Anthocyanins are found in flowers, stems, leaves, and fruits of most plants [11,12,13,14], and it is conducive to plant response to biotic and abiotic stresses such as low temperatures, drought, disease, ultraviolet light, pests, and so on [12,15,16]. In nature, they are mainly bound to sugars by glycosidic bonds and form of anthocyanin, including cyanidin, pelargonidin, delphinidin, peonidin, petunidin, and malvidin [17], which all contain the basic framework structure of C6-C3-C6 [9] and special glycosyl modification [9,12].

Anthocyanin biosynthesis pathway in higher plants mainly consists of three parts. Firstly, 4-Coumaroyl-CoA was synthesized by phenylalanine under continuous catalysis of phenylalanine ammonia-lyase (PAL, EC:4.3.1.24), 4-coumarate-CoA ligase (4CL, EC:6.2.1.12), and trans-cinnamate 4-monooxygenase (CYP73A, EC:1.14.14.91); then, 4-Coumaroyl-CoA was catalyzed by chalcone synthase (CHS, EC:2.3.1.74) and chalcone isomerase (CHI, EC:5.5.1.6) to produce naringenin, the different dihydroflavonols were synthesized under the action of 3-dioxygenase (F3H, EC:1.14.11.9), flavonoid 3′-monooxygenase (F3′H, EC:1.14.14.82), and flavonoid 3′,5′-hydroxylase (F3′5′H, EC:1.14.14.81). Finally, the different anthocyanins were generated under the catalysis of dihydroflavonol 4-reductase (DFR, EC:1.1.1.219), anthocyanidin synthase (ANS, EC:1.14.20.4), and anthocyanidin 3-O-glucosyltransferase (BZ1, EC:2.4.1.115). CHS is widely present in plants, and its protein sequences are highly conserved. Several studies have shown that the expression of the CHS gene plays an important role in the regulation of anthocyanin accumulation [18,19,20]. CHI is a key rate-limiting enzyme in the anthocyanin synthesis pathway, and its activity has a crucial effect on the accumulation of chalconesits [21]. F3H, F3′H, and F3′5′H also significantly affect the formation of anthocyanins to participate in the regulation of plant flower color changes [22]. DFR is a key enzyme in the downstream of the anthocyanin synthesis to regulate the production of flower color. It can catalyze dihydrokaempferol, dihydroquercetin, and dihydromyricetin to produce colorless anthocyanins, pelargonidin, cyanidin, and delphinidin, respectively [23]. *DFR* gene widely exists in a variety of plants and has been identified in *Arabidopsis thaliana* (1), *Nicotiana tabacum* (2), *Petunia hybrida* (3), *Ginkgo biloba* (3), *and Vitis vinifera* (3).

*Bletilla striata* (Thunb. ex Murray) Reichb. f. (*B. striata*) is a perennial herb of the *Bletilla* genus in the Orchidaceae, which is often used as medicine with dry pseudobulb [24]. As an orchid, it is also of significant ornamental value and has beautiful flowers with different colors, generally including purple, purplish red, pink, or light pink. However, there was little research about flower color in *B. striata*, and only the CHS gene family had been identified, which was found to be involved in flavonoid and anthocyanin synthesis [25], but with scant understanding of DFR. Studies on other orchid species have shown that *DFR* is expressed in the purple region of flowers in *Bromheadia finlaysoniana* and is the main enzyme gene responsible formation of purple spots in *P. aphrodite*, but the expression of *DFR* in the sepals of other varieties like ’Panda’ showed no significant difference [26]. This might be caused by the action of different pigment-synthesizing enzymes leading to color formation. Therefore, it is particularly important to predict the anthocyanin synthesis pathway, identify related enzyme genes, and understand the role of *DFR* in the formation of flower color in *B. striata.* Moreover, many studies have found the involvement of MYB transcription factors in regulating anthocyanin synthesis and affecting gene expression of related enzymes in plants. *NnMYB5* regulates petal anthocyanin accumulation to influence petal color [27]. In the *spiny Solanum*, the natural variation in the MYB binding sites within the promoter region of the *DFR* gene and the selective expression of *DFR* affected anthocyanin accumulation [28]. In *Herba epimedii* (Epimedium), EsMYBA1 can interact with several bHLH regulators of the flavonoid pathway and activate the promoters of *DFR* [29]. The *FeMYBF1* in *F. esculentum* was also found to promote the accumulation of flavonoids by activating the *DFR*, *FLS*, and *CHS* promoters [30].

In this study, the petals of pink flowers (designated as the WP group) and purple flowers (P group) were collected from different locations in Shaanxi Province for the determination of total anthocyanins and total flavonoids, and isolated and detected the anthocyanin components. The DNA was extracted from the petals of the P and WP groups, and the resequencing analysis was performed separately to identify the variation of different samples. The anthocyanin synthesis pathway was analyzed based on the genomic database of *B. striata* and the KEGG database. The key enzyme genes were identified, and the variant genes related to anthocyanin biosynthesis were screened and analyzed. In addition, the further regulatory function investigation of *BsDFR4* on flower color formation was completed through its heterologous overexpression in *P. aphrodite* by Agrobacterium-mediated transient transformation. Finally, the interaction between BsMYBs and the promoter of *BsDFR4* was analyzed. This study contributes to a more effective understanding of the causes of *B. striata* flower color and the differences in anthocyanin biosynthesis among different flower colors, which provides a reference for the study of orchid flower color.

## 2. Results

### 2.1. B. striata with Purple Flower Have Higher Content of Anthocyanin and Flavonoid

**Figure 1:**
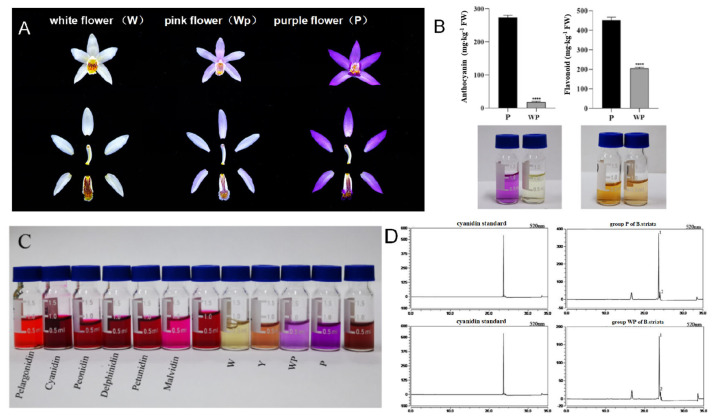
The different flower colors of *B. striata* and the analysis of flavonoids and anthocyanins in flowers. (**A**), the flowers with different colors. (**B**), the total anthocyanins contents and total flavonoids contents of *B. striata* flower with purple (P) and pink (WP), the data are means ± standard deviation (*n* = 3), **** representing *p* < 0.001; (**C**), comparison of flower extracts of *B. striata* with different colors, W representing white flowers, Y representing yellow flowers; (**D**), HPLC analysis of anthocyanin components in purple and pink flowers.

In order to explore the differences between *B. striata* with different flower colors, we collected the plant with pink flowers (WP group) and purple flowers (P group) for the determination of total flavonoids and total anthocyanins (Figure 1A). The WP group had a total flavonoid content of 205.07 ± 0.65 mg/kg, while the P group had 451.33 ± 1.27 mg/kg (*p* < 0.01), which was 2.20 times greater than the WP group. The total anthocyanin content of the WP group was 18.00 ± 0.26 mg/kg, and the P group was 274.00 ± 0.54 mg/kg (*p* < 0.01), which was about 15.22 times higher than the WP group. Therefore, we speculated that the anthocyanin concentration may be a key factor in the variation in flower colors.

Further, we separated and identified the anthocyanin components of *B. striata* petals using HPLC to investigate the elements contributing to this variation. The peaks of groups P and WP were consistent with the cyanidin standard, which both reached the maximum peak height at about 23.65 min. The contents of cyanidin were 0.34 mg/mL (group P) and 0.15 mg/mL (group WP) (*p* < 0.01), respectively. This suggested that cyanidin was the primary component in the flower of *B. striata*. Therefore, we believe that the amount of cyanidin in the *B. striata* flower was a crucial factor in the relationship between its various colors. However, the absorption peak in the results is accompanied by a shoulder peak, indicating there are other components that have not yet been fully isolated from the sample.

### 2.2. More Variations Are Observed in B. striata with Pink Flowers

**Figure 2:**
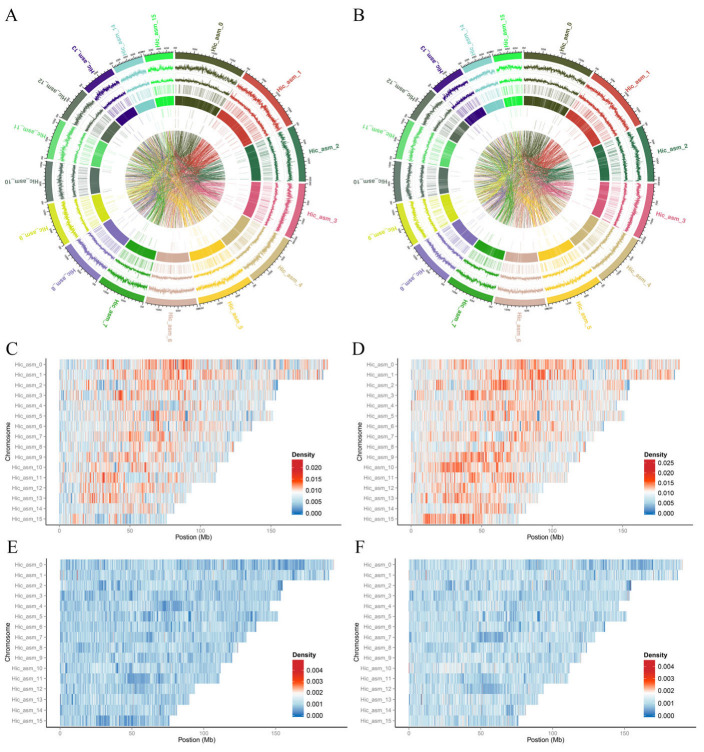
Resequencing analysis of *B. striata* flower with purple and pink. (**A**) (purple flower) and (**B**) (pink flower), the distribution map of genome structure variation, from outer to inner circle are chromosome name, SNP variation, small fragment insertion and deletion, copy number duplication, copy number deletion, chromosome insertion, chromosome deletion, and chromosome inversion; (**C**) (purple flower) and (**D**) (pink flower), heat maps of SNP distribution on different chromosomes; (**E**) (purple flower) and (**F**) (pink flower), InDel distribution heat map on different chromosomes.

In order to investigate the molecular mechanisms of the formation of different flower colors in *B. striata*, the resequencing analysis was performed using the Illumina sequencing platform based on the genome database of our laboratory, and the results of the P group and WP group are shown as visual genomic structural variation distribution maps (Figure 2A,B). A total of 137.32 Gb data was generated, Q20 was above 96.95%, Q30 was below 91.57%, GC content was between 35.99% and 36.58% (Table S1), the ratio to the reference genome was between 94.41% and 97.28%, and the average coverage depth was between 22.71× and 26.40× (Table S2). The sequencing is of high quality and can be applied to further research.

In order to clearly display the distribution of SNPs and InDels on chromosomes, using a 100 kb window size to calculate the density of SNPs and InDels in each window, some heatmaps were created to show the distribution of SNPs and InDels on the chromosomes (Figure 2C–F). According to the resequencing results, a total of 18,094,102 SNPs, 2,163,644 InDels, 100,387 SVs, and 74,672 CNVs were found in the P group samples, including 130,462, 7213, 10,562, and 3614 of variants located in exon regions, respectively. A total of 23,007,948 SNPs, 2,390,821 InDels, 105,919 SVs, and 61,315 CNVs were found in the WP group samples, in which 177,565, 8347, 9218, and 4078 variants in exon regions, respectively (Table 1, Tables S2 and S3, Figure S1). SNP was the main type of variation between different samples, and the variation in the WP was significantly more than that in the P group, while the CNV of variants in the WP was significantly less than that in the P group. Thymine–adenine > cytosine–guanine (T:A > C:G) was the most frequent SNP variant in both samples, followed by cytosine–guanine > thymine–adenine (C:G > T:A), while cytosine–guanine > guanine–cytosine was the least frequent variant (Figure S2).

### 2.3. Prediction of Anthocyanin Synthesis Pathway in B. striata

**Figure 3:**
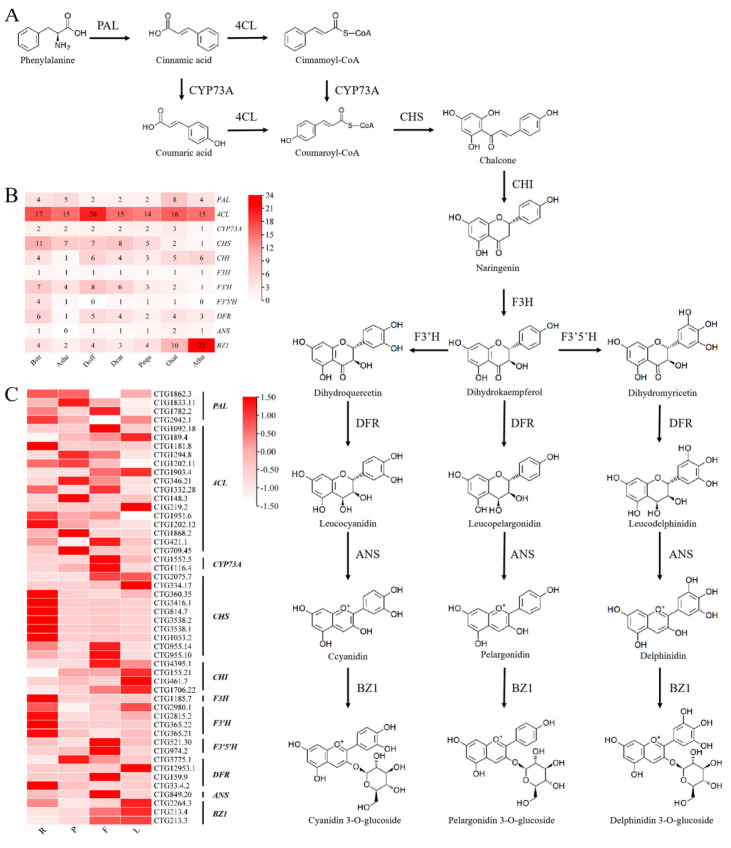
Analysis of anthocyanin synthesis pathway and expression pattern of key enzyme genes in *B. striata*. (**A**), anthocyanin synthesis pathway and related enzymes, PAL, phenylalanine ammonialyase; 4CL, 4-coumarate-CoA ligase; CYP73A, trans-cinnamate 4-monooxygenase; CHS, chalcone synthase; CHI, chalcone isomerase; F3H, Naringenin 3-dioxygenase; F3 ’h, flavonoid 3′-monooxygenas, F3′5′H, flavonoid 3′5′-hydroxylase; DFR, dihydroflavonol 4-reductase; ANS, anthocyanidin synthase; BZ1, anthocyanidin 3-O-glucosyltransferase. (**B**), the number of related enzyme genes in different species, including *A. thaliana* (Atha), *O. sativa* (Osat), *B. striata* (Bstr), *P. equestris* (Pequ), *A. shenzhenica* (Ashe), *D. officinale* (Doff), and *D. catenatum* (Dcat). (**C**), the expression patterns of anthocyanin synthesis-related genes in different tissues of *B. striata*, R, P, F, and L represent roots, pseudobulbs, flowers, and leaves. The relative expression of genes is represented by the intensity of color in each region. Higher values are shown in red, while lower values are shown in white.

According to the KEGG database, the anthocyanin synthesis pathway of *B. striata* was analyzed and predicted. All relevant enzyme genes were screened and identified based on our genomic database (Figure 3A). A total of 61 genes were identified, including 17 genes encoding 4CL (4-coumarate-CoA ligase), 11 genes encoding CHS (chalcone synthase), 1 gene encoding F3H (Naringenin 3-dioxygenase), 4 genes encoding F3′5′H (flavonoid 3′,5′-hydroxylase), and 6 genes encoding DFR (dihydroflavonol 4-reductase). The CHS, F3′H, F3′5′H, and DFR gene families were expanded, the BZ1 families were contracted, while 4CL and F3H remained stable. Further, compared with *A. thaliana* and *O. sativa*, the number of these enzyme genes in orchidaceae remained relatively stable (Figure 3B, Table S4).

**Figure 4:**
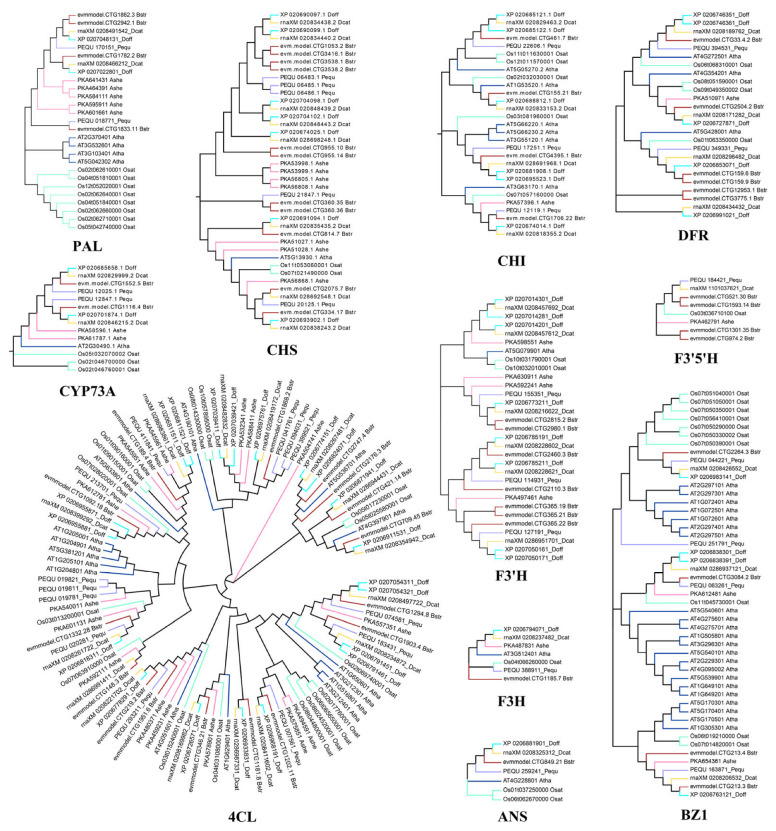
Phylogenetic trees of key enzyme genes in anthocyanin synthesis pathway in 7 species. The phylogenetic trees were constructed using MEGA 7.0 software with the neighbor-joining method under 1000 bootstrap replicates. Different branch colors indicate the different species, including blue (*A. thaliana*, Atha), light green (*O. sativa*, Osat), dark red (*B. striata*, Bstr), purple (*P. equestris*, Pequ), pink (*A. shenzhenica*, Ashe), light blue (*D. officinale*, Doff), and yellow (*D. catenatum*, Dcat).

The results of expression patterns in various tissues showed that the different genes encoding the same enzyme exhibited tissue-specific expressions. The four genes encoding PAL were highly expressed in roots, pseudobulbs, leaves, and flowers, respectively; the expression of two genes encoding CYP73A and two genes encoding F3′5′H were significantly higher in flowers, three genes encoding BZ1 had the highest expression levels in leaves; multiple genes encoding CHS were highly expressed in roots while encoding CHI exhibited higher expression levels in leaves (Figure 3C). The phylogenetic trees of different gene families in the anthocyanin synthesis pathway were constructed to elucidate the evolutionary relationship (Figure 4).

### 2.4. Multiple Anthocyanin Pathway Enzyme Genes Are Mutated

**Figure 5:**
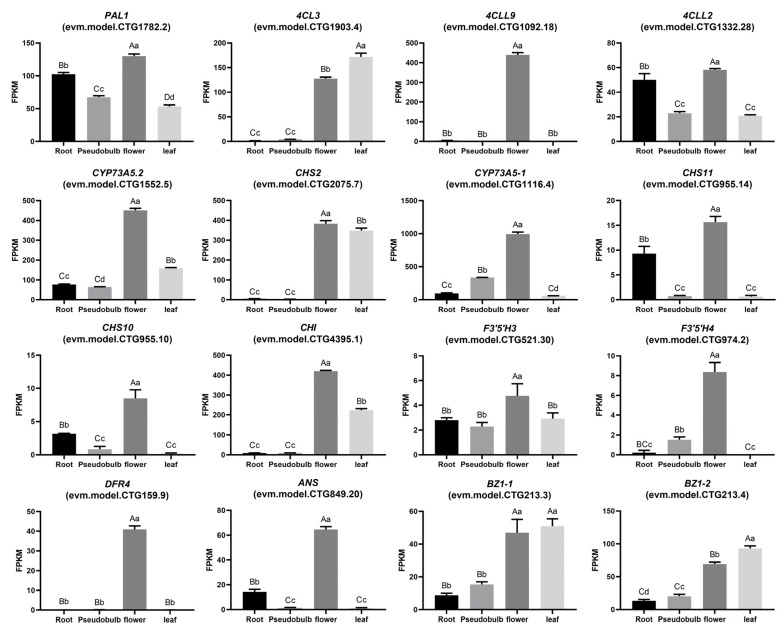
Expression profile of 16 genes highly expressed in flowers of *B. striata.* Data are means ± standard deviation (*n* = 3). Different lowercase letters (a, b, c, d) indicate a significant difference at *p* ≤ 0.05, whereas different uppercase letters (A, B, C, D) indicate a significant difference at *p* ≤ 0.01.

There were 16 genes highly expressed in flowers of *B. striata* were screened to analyze the variation of enzyme genes (Figure 5), and the analysis focused on the variants occurring in the exonic region. The results showed that all genes had SNP mutations in exons (Table 2, Tables S5 and S6). There were more SNP variations in the WP group than in the P group, and the synonymous SNV is significantly more than the nonsynonymous SNV. Specifically, evm.model.CTG1782.2 (encoding PAL) had the largest number of SNP variants, with 48 and 50 SNP variants in the P group and WP group, including 18 and 17 nonsynonymous SNVs, respectively. There were 18 synonymous SNVs and 12 nonsynonymous SNVs shared by groups P and WP, while groups P and WP had 6 and 5 unique nonsynonymous SNVs, respectively. evm.model.CTG1903.4 (encoding 4CL) had 13 and 23 SNP variants in the P group and WP group, while evm.model.CTG2075.7 (encoding CHS) had 8 and 12 SNP variants and evm.model.CTG849.20 (encoding ANS) had 6 and 9 SNP variants. Furthermore, evm.model.CTG1782.2 (encoding PAL), evm.model.CTG1903.4 (encoding 4CL), and evm.model.CTG159.9 (encoding DFR) had the variants of stop gain in the WP group. *BsPAL1* (evm.model.CTG1782.2) exhibited significant expression across multiple tissues, *Bs4CL3* (evm.model.CTG1903.4) showed prominent expression in both flowers and leaves, while *BsDFR4* was exclusively and highly expressed in flowers (Figure 5). We speculated that it may be the reason for the different flower colors of *B. striata*.

Moreover, only five genes (evm.model.CTG1092.18 and evm.model.CTG1903.4 encoding 4CL, evm.model.CTG4395.1 encoding CHI, evm.model.CTG974.2 encoding F3′5′H, and evm.model.CTG213.3 encoding BZ1) occurred in the InDel variants. Compared with group P, a unique frameshift insertion of the C base occurred in evm.model.CTG974.2 in group WP, while a non-frameshift deletion occurred in evm.model.CTG4395.1. In contrast to SNP and InDel variants, the SV variations in the exon region of these 16 genes were significantly higher in group P than in group WP (Table S5).

### 2.5. BsDFR4 Overexpression Alters the Petals Color of P. aphrodite and Affects the Expression of Color-Related Genes

**Figure 6:**
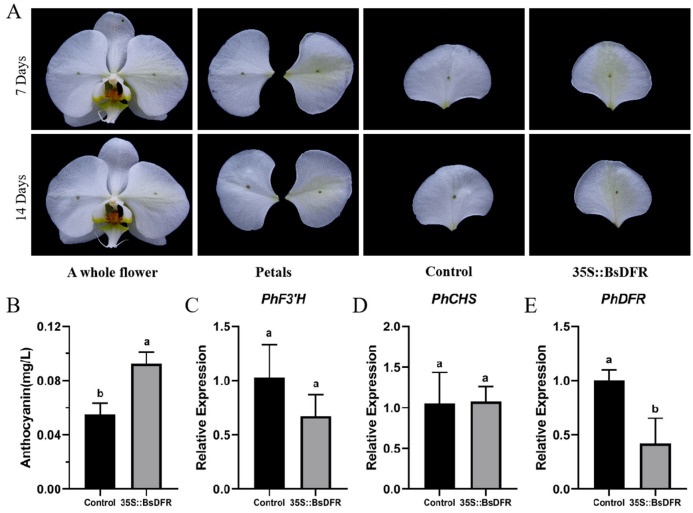
*BsDFR4* heterologous overexpression in *P. aphrodite* and expression analysis. (**A**), the phenomenon of color change in *P. aphrodite*. (**B**), the anthocyanins content of *P. aphrodite* petals. (**C**), expression analysis of *PhF’3H*. (**D**), expression analysis of *PhCHS*. (**E**), expression analysis of *PhDFR*. Data are means ± standard deviation (*n* = 3). Different lowercase letters (a, b) indicate a significant difference at *p* ≤ 0.05.

To further investigate the function of *BsDFR4* in the anthocyanin biosynthesis pathway, the transient overexpression of *BsDFR4* in the petals of *P. aphrodite* with white flowers was conducted using Agrobacterium-mediated transformation. The changes in petal color were observed at 7 days and 14 days post-transformation (Figure 6A). After 7 days of cultivation, the petals began to change from white to pale yellow. After 14 days of cultivation, overexpressed petals gradually deepened in color, turning a distinct yellow-green. The petals of the experimental and control groups were subjected to anthocyanin content determination and qRT-PCR analysis (Figure 6B–E). The petals of the experimental group demonstrated a significantly higher anthocyanin content compared to those of the control group (Figure 6B). Compared to the control group, the expression level of *PhDFR* was downregulated in the experimental group (*p* < 0.05), while the expression of *PhF’3H* and *PhCHS* showed no significant difference. Overexpression of *BsDFR4* in *P. aphrodite* petals might inhibit the expression of *PhDFR*, thereby influencing the color change from white to yellow-green in the petals.

### 2.6. BsMYB36 and BsMYB51 Can Bind Directly to the Promoter Region of BsDFR4

**Figure 7:**
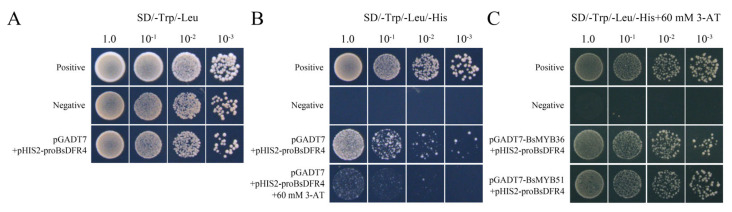
Yeast transactivation activity assay and yeast one-hybrid (Y1H) assay. (**A**,**B**), yeast transactivation activity assay of *proBsDFR4*, the yeast colonies were cultured on SD/-Trp/-Leu medium (**A**) and were screened on SD/-Trp/-Leu/-His medium (**B**). (**C**), yeast one-hybrid (Y1H) assay between *proBsDFR4* and *BsMYB36* (and *BsMYB51*), the yeast colonies were screened on SD/-Trp/-Leu/-His medium added 60 mM 3-AT. The positive control was pGADT7-p53 + pHIS2-p53, and the negative control was pGADT7 + pHIS2-p53.

MYB transcription factors can participate in the accumulation of anthocyanins by affecting related enzymes in the anthocyanin synthesis pathway. *BsMYB36* and *BsMYB51* belong to the S7 and S5 subfamily, which are involved in the regulation of flavonoids and anthocyanin. On the SD/-Trp/-Leu medium, positive control, negative control, and the recombinant plasmids pGADT7 + pHIS2-*ProBsDFR4* grew normally (Figure 7A). However, on the SD/-Trp/-Leu/-His medium, yeast transformed with the negative control failed to grow, while the positive control and pGADT7 + pHIS2-*ProBsDFR4* grew normally, and the growth was significantly inhibited by the addition of 60 mM 3-AT (Figure 7B). These results indicated that the promoter of *BsDFR4* possessed transcriptional self-activation activity and could be effectively inhibited by 60 mM of 3-AT. According to the results of the yeast one-hybrid assay, it was observed that the positive and the two experimental transformants (pGADT7-*BsMYB36* + pHIS2-*ProBsDFR4* and pGADT7-*BsMYB51* + pHIS2-*ProBsDFR4*) were able to grow on the SD/-Trp/-Leu/-His medium containing 60 mM 3-AT, while the negative control failed to grow (Figure 7C). This suggested that both *BsMYB36* and *BsMYB51* could bind to the *BsDFR4* promoter through MYB binding elements, potentially influencing the expression of *BsDFR4*.

## 3. Discussion

The flower color is one of the important characteristics of petals in flowering plants, which is usually closely related to the type, concentration, and distribution of anthocyanin [2]. In this study, the total flavonoid and anthocyanin contents in purple flowers of *B. striata* were significantly elevated compared to those in pink flowers (approximately 2.20-fold and 15.22-fold higher, respectively, *p* < 0.01), confirming that darker pigmentation correlates with anthocyanin accumulation.

Given the diversity of anthocyanin types contributing to plant coloration, the specific anthocyanins in *B. striata* petals were analyzed. Cyanidin was identified as the predominant component, aligning with prior reports that cyanidin, pelargonidin, and peonidin are major anthocyanins in orchids [31,32]. Cyanidin is the main component of anthocyanins in mulberry, providing red, purple, and black purple to plant tissues, which could make rose germplasm appear red, pink, and purplish red. The cyanidin 3-O-rutinoside and peonidin 3-O-rutinoside were also found as the major anthocyanins in *Cymbidium* orchid [33], while delphinidin and cyanidin are key pigments in violet Epimedium species [34]. Notably, cyanidin concentrations differed significantly between groups P (0.34 mg/mL) and WP (0.15 mg/mL), suggesting its pivotal role in color variation. However, shoulder peaks in absorption spectra indicated uncharacterized components, implying incomplete isolation of anthocyanins in *B. striata*.

Resequencing analyses revealed a substantial genomic divergence between purple (group P) and pink (group WP) flowers. Compared to the reference genome (derived from purple-flowered B. striata), group WP exhibited significantly higher SNP (Single-Nucleotide Polymorphism) variation in exonic, intronic, upstream, and downstream regions, as well as in transition/transversion ratios. The heterozygosity rates of SNPs (number of heterozygous SNPs/genome size) were 4.332‰ (group P) and 7.931‰ (group WP), validating sequencing accuracy (Table 1). Group WP also displayed increased InDel (Insertion–Deletion) frequencies but fewer CNVs (Copy Number Variations) relative to group P, while SV (structural variation) counts were comparable (Table S3).

A total of 61 genes encoding 11 key enzymes in the anthocyanin biosynthesis pathway were identified in *B. striata*. There were more encoding genes related to anthocyanin synthesis. Comparative genomic analyses with other orchids (*P. equestris*, *A. shenzhenica*, *D. officinale*, and *D. catenatum*) revealed expansion of most gene families in *B. striata*, except for CYP73A, F3H, ANS, and BZ1, which remained conserved (Figure 3B). The number of gene family members in *D. officinale* and *D. catenatum* was more similar to that in *B. striata*, which might be due to their closer evolutionary relationship [35]. Expression profiling demonstrated functional redundancy among paralogs, with genes encoding identical enzymes exhibiting analogous tissue-specific expression patterns in *B. striata* (Figure 3C).

According to the expression profile of all genes, 16 genes highly expressed in flowers were screened to analyze the variation of genes related to flower color formation. There were 131 SNP variants located in exon regions in group P and 164 in group WP, with four and six InDel variants, respectively (Table S5). In the upstream part of the anthocyanin pathway, evm.model.CTG1782.2 encoding PAL, evm.model.CTG1092.18, evm.model.CTG1903.4, and evm.model.CTG1332.28 encoding 4CL all had different degrees of unique SNP variations in group P and group WP. In particular, evm.model.CTG1782.2 and evm.model.CTG1903.4 separately had an SNP variation that exonic stop codon gain (Table 2 and Table S6). These might affect the accumulation of flavonoids and anthocyanins in *B. striata* flowers with different colors [36]. The CHS was reported to be involved in the regulation of anthocyanin accumulation and affects color formation in apples and strawberries [19,20]. According to the results, three CHS genes (evm.model.CTG2075.7, evm.model.CTG955.10 and evm.model.CTG955.14 were found to have unique nonsynonymous SNPs in group WP and group P, respectively (Table 2). Furthermore, evm.model.CTG4395.1 had one unique nonsynonymous SNPs in group WP, respectively. As a key rate-limiting enzyme, CHI will quickly convert chalcone into naringin under normal conditions, and the flower color changes when CHI activity is inhibited. For example, silencing the CHI gene in tobacco, the flower color was changed to yellow [21]. We believe that these may be closely related to the formation of different colors in *B. striata*. Moreover, F3′5′H was another important enzyme effecting the anthocyanin accumulation and the color formation. It was reported that overexpression of the *Saintpaulia ionantha F3′5′H* gene in tobacco and the *Aconitum carmichaeli F3′5′H* gene in petunia caused obvious changes in flower color [22]. Similar phenomena have been also reported in *Epimedium sagittatum* [34,37]. In this study, we found that evm.model.CTG521.30 encoding F3′5′H had three unique SNP variants in group WP, while evm.model.CTG974.2 occurred in six unique SNPs in group P and five in group WP (Table 2). This might be one of the reasons that affect the formation of different flower colors and the accumulation of anthocyanins in *B. striata*. Collectively, these findings underscore the interplay of cyanidin accumulation, genomic structural variation, and diversity in anthocyanin pathway genes as determinants of flower color in *B. striata*.

In the downstream phase of anthocyanin biosynthesis, a variant of the *DFR* gene (evm.model.CTG159.9) was identified in *B*. *striata*, characterized by a premature termination codon resulting from a mutation at the 145th base of the coding sequence. DFR catalyzes the conversion of dihydroflavonols to colorless anthocyanins for anthocyanin and proanthocyanidin biosynthesis (Table 2). Orthologs of *DFR* have been functionally characterized in *Brassica napus* [38], *Ginkgo biloba* [39], and *Vitis vinifera* [40]. Heterologous overexpression of *MaDFR* in tobacco increased anthocyanin accumulation and intensified floral pigmentation to deepen the flower color [41], while analogous experiments with *Gerbera jamesonii DFR* [42] and *G. biloba DFR* [43] yielded similar phenotypic outcomes. Conversely, *DFR* suppression in *Petunia* induced color lightening (from purple to pale pink) or complete whitening [44,45], and *dfr* mutants exhibited white or pale yellow pigmentation [43]. These collectively implicate evm.model.CTG159.9 as a critical regulator of flower color in *B. striata*, consistent with the established role of *DFR* in pigmentation processes across species, including *Brassica napus* [46]. To elucidate the regulatory mechanism of *BsDFR4* in floral coloration, heterologous overexpression experiments were conducted in *P*. *aphrodite*. It was found that overexpression of *BsDFR4* (35S::*BsDFR4*) is a shift from white to yellow-green petals. The qRT-PCR analysis focused on three relative genes involved in the anthocyanin glycoside synthesis pathway of *P. aphrodite*, including *PhCHS*, *PhF3′H*, and *PhDFR*. The results revealed significant downregulation of *PhDFR* (*p* < 0.05) in experimental groups, while *PhCHS* and *PhF3′H* expression remained unaffected (Figure 6B–D). This suggested that the pigmentation and the deepening of color in *P*. *aphrodite* petals were indeed attributable to the overexpression of *BsDFR4*.

As pivotal regulators of plant secondary metabolism, MYB transcription factors frequently modulate flower coloration by targeting key genes within the anthocyanin biosynthesis pathway, including *CHS*, *F3′H*, *DFR*, *ANS*, and *UFGT*. For example, *AtMYB75/PAP1* enhances anthocyanin accumulation in *Arabidopsis* by directly binding to the *DFR* promoter [47]. Conversely, *FtMYB18* negatively regulates *CHS* and *DFR* expression, thereby influencing the biosynthesis of anthocyanins and proanthocyanidins [48]. Notably, two MYB subfamilies, S5 and S7, have been identified as central players in the regulation of anthocyanin and flavonoid biosynthesis. Members of the S7 subfamily, such as *AtMYB11*, *AtMYB12*, and *AtMYB111*, are known to govern flavonol production [49], while S5 subfamily members, including *MdMYB9* and *MdMYB11*, regulate anthocyanin and proanthocyanidin accumulation in apple (*Malus domestica*) [50]. In this study, we identified *BsMYB51* (S5 subfamily) and *BsMYB36* (S7 subfamily) as direct transcriptional regulators of *BsDFR4*, demonstrating their ability to bind to the *BsDFR4* promoter region. This finding suggested that the formation of flower color in *B*. *striata* might depend on the coordinated regulatory actions of specific MYB subfamily members. These results provided critical insights into the transcriptional regulatory networks underlying flower color formation and laid a foundation for further mechanistic studies in *B. striata* and related species.

## 4. Materials and Methods

### 4.1. Collection of Plant Materials

In this study, the experimental plants were conducted from Huyi, Lueyang, Fengxian, and Yangxian, Shaanxi Province, which were the main distribution areas of *B. striata* in China. The different color flowers of *B. striata* were picked and collected during the blooming period (from mid-April to mid-May). The petals of the pink flower (designated as the WP group) and the purple flower (designated as the P group) were collected separately, and the different flower colors were taken from at least three healthy plants. The samples were flash-frozen with liquid nitrogen following collection before storage at −80 °C.

### 4.2. Separation and Determination of Anthocyanin Content and Components

The flower petals were immersed in extract (1:1, 0.1% HCl:95% CH_3_CH_2_OH [*v*:*v*]), sonicated (30 min, 60 W), centrifuged (1200× *g* rpm, 15 min), and the supernatant was extracted and diluted 10-fold. UV–Vis spectrophotometry was used to obtain the total anthocyanin content of the samples with some modifications concerning [51]. The absorbance value of the diluted extract was measured at 520 nm, and the total anthocyanin content in the petal samples was calculated from the standard curve made using the standard. Sodium nitrite aluminum chloride colorimetry was selected as the measurement method of total flavonoids [52]. The ethanol (60%) is selected as the standard control, and the extract of methanol and acetone is used as the sample blank control. The absorbance value is measured at 510 nm, and the content of total flavonoids in the sample is calculated through the standard curve.

The petal extract was dissolved in CH_3_COOH-CH_3_OH (85:15:0.5, CH_3_OH–water–CH_3_COOH [*v*:*v*:*v*]). The mixed solution was extracted by sonication (100 W, 10 min) and centrifuged (1000× *g* rpm, 2 min). The supernatant was further sonicated for 10 min, centrifuged for 1 min, and filtered through a membrane. High-Performance Liquid Chromatography (Thermo Fisher Scientific, Waltham, Massachusetts, USA) and a C18 column were used to analyze the extracted anthocyanins to determine their type and composition [38]. Chromatograms were acquired at 520 nm. Eluent A was chromatographic-grade methanol solution, eluent B was 0.1% phosphoric acid aqueous solution. A gradient elution protocol as follows was used: 65% B at 0 min, 65% B at 20 min, 15% B at 22 min, 65% B at 28 min, and 65% B at 35 min. The flow rate was 0.8 mL min^−1^, and the column temperature was maintained at 35 °C.

### 4.3. Resequencing Analysis of B. striata with Different Flower Color

The genomic DNA of the different flowers was extracted and the purity and concentration were determined by using a spectrophotometer (Thermo Fischer Scientific, Waltham, Massachusetts, USA) to obtain the high-quality DNA extracts. The resequencing analysis was carried out on samples with different color flowers using the Illumina sequencing platform by Novogene Co., Ltd. (Beijing, China). Based on the *B. striata* genome database of our laboratory, further analyses were performed. Quality control of the raw sequences was performed using fastp 0.20.0, followed by alignment of the data to the reference genome using BWA 0.7.8 [53]. The SNPs and InDels variants were detected using SAMTOOLS 1.3.1 [54], while structural variations (SVs) were identified using BreakDancer 1.4.4 [55]. Copy number variations (CNVs) were analyzed using CNVnator V0.3 [56], and the annotated structure and function of the variants were determined using ANNOVAR (2015Dec14) [57].

### 4.4. Prediction of Anthocyanin Synthesis Pathway and Identification of Enzyme Genes in B. striata

Referring to KEGG annotation information, the anthocyanin synthesis pathway was analyzed and predicted, and the key enzyme genes in the pathway were identified based on the genome database of *B. striata*. The amino acid sequences of all enzyme genes in *A. thaliana*, *O*. *sativa*, *P. equestris*, *A. shenzhenica*, *D. officinale*, and *D. catenatum* were obtained from the TAIR database (https://www.arabidopsis.org/ (accessed on 27 March 2023)), the JGI Phytozome website (https://phytozome.jgi.doe.gov/pz/portal.html (accessed on 27 March 2023)), NCBI database (https://www.ncbi.nlm.nih.gov/gene/ (accessed on 27 March 2023)), and KEGG database (https://www.kegg.jp/kegg/pathway.html (accessed on 27 March 2023)), respectively. The neighbor-joining (NJ) phylogenetic trees were constructed using MEGA software (with 1000 bootstraps) to analyze the evolutionary relationships, respectively. Combined with the transcriptome analysis in different tissues of *B. striata*, we analyzed the expression patterns of these enzyme genes.

### 4.5. Screening of Variation Genes Related to Flower Color Formation of B. striata

According to the expression patterns of enzyme genes in the anthocyanin synthesis pathway, those genes specifically expressed or significantly highly expressed in flowers were screened. The type and number of variants in these genes were analyzed based on the results of the resequencing analysis. The analysis focused on the SNP and InDel variants, and the share and unique variants in groups P and WP were counted to evaluate the differences in different flowers.

### 4.6. BsDFR4 Heterologous Overexpression and Expression Analysis of P. aphrodite

To further investigate the function of the *BsDFR4*, this study utilized *A.tumefaciens* GV3101 for transformation experiments on the petals of *P. aphrodite* with white flowers. Firstly, the full-length CDS of *BsDFR4* was cloned into the TOPO vector. Then, it was constructed into a pDONR207 vector using a BP recombination reaction and was eventually cloned into pEarleyGate202 using an LR recombination reaction of the Gateway technology (Invitrogen, Waltham, Massachusetts, USA). The recombinant vector pEarleyGate202-*BsDFR4* and the empty vector pEarleyGate202 were separately transformed into Agrobacterium. Positive colonies were selected and cultured to inject the symmetric petals of the same flower. The detailed injection procedures referred to the research about studying flower color in *Cymbidium ensifolium* [58]. The transformed *P. aphrodite* was placed and cultivated at 25 °C with a 16 h light and 8 h dark. The color change of the petals was observed during each photoperiod. The circular samples with a diameter of 2 cm centered around the injection site on the petals were collected. Then, the anthocyanin content in the samples was determined via the pH differential method [59]. The samples were grounded and suspended in potassium chloride–hydrochloric acid (KCl–HCl) buffer (pH = 1.0) and sodium acetate buffer (pH = 4.5), respectively. After centrifugation at 3000× *g* rpm for 5 min, the supernatant was collected and equilibrated for 15 min, and the absorbance was measured at wavelengths of 530 nm and 700 nm. The concentration was calculated using the formula: C (mg/L) = (∆A × 449.2 × sample dilution factor × 1000)/(26,900 × 1) where ∆A is (A_530_ − A_700_)_pH 1.0_ − (A_530_ − A_700_)_pH 4.5_. The total RNA was extracted, and the cDNA was synthesized. The expression of genes involved in the anthocyanin biosynthesis pathway was analyzed by the qRT-PCR, including *PhF3′H*, *PhCHS*, and *PhDFR*. Primer sequences are detailed in Table S7.

### 4.7. Yeast Transactivation Activity Assay and Yeast Tone-Hybrid (Y1H) Assay

The promoter region of *BsDFR4* was cloned based on the gene sequences of *B. striata* (Table S8, Figure S3). The *ProBsDFR4* was fused to pHIS2 to generate pHIS2-*ProBsDFR4* using *EcoRI* and *MluI*. The pGADT7 and pHIS2-*ProBsDFR4* were co-transformed into yeast strain Y187. The positive yeast colonies were cultured on SD/-Leu/-Trp medium and screened on SD/-Leu/-Trp/-His medium to analyze the transactivation activity of *ProBsDFR4* at 28 °C for 4 days. The *BsMYB36* (from the S7 subfamily of R2R3-MYBs) and *BsMYB51* (from the S5 subfamily of R2R3-MYBs) were cloned based on the genome database of *B.striata*. Then, the pGADT7-*BsMYB36* and pGADT7-*BsMYB51* recombinant vectors were constructed using BP and LR recombination of Gateway technology. Finally, pGADT7-*BsMYB36* and pGADT7-*BsMYB51* were co-transformed with pHIS2-*ProBsDFR4* into the yeast strain Y187, respectively. The positive colonies were cultured on SD/-Leu/-Trp medium and screened on SD/-Leu/-Trp/-His medium to analyze the interactions between *proBsDFR4* and BsMYBs.

## 5. Conclusions

In this study, we collected flowers with different colors from *B. striata* and examined their anthocyanin contents and components. The results showed that purple flowers had significantly higher levels of anthocyanins compared to pink flowers, with cyanidin being the main anthocyanin in *B. striata*. Subsequently, we performed a resequencing analysis to explore the molecular basis of different flower color formations in *B. striata*. We found a total of 18,094,102 and 23,007,948 SNP variations in purple and pink flowers, respectively. Based on the genomic databases of *B. striata*, we identified 61 genes involved in the anthocyanin synthesis pathway, with an increased number of gene family members. Among these genes, we found 16 significantly highly expressed genes in flowers, with 131 and 164 SNP variants located in the exon regions of purple and pink flowers, respectively, including 48 and 62 nonsynonymous SNVs. One of the genes encoding *DFR* acquired a termination codon in advance, resulting in the protein being unable to be synthesized normally. The heterologous overexpression of *BsDFR4* in *P. aphrodite* by Agrobacterium-mediated transient transformation changed petal color from white to yellow-green, demonstrating that *BsDFR4* indeed played a regulatory role in the formation of flower color. Subsequently, we will further attempt to establish the corresponding genetic transformation system in *B. striata*, thereby conducting in-depth research on the functional characterization of *BsDFR4*. Meanwhile, whether other genes in the anthocyanin synthesis pathway play significant roles in the floral pigmentation of *B. striata* remains a critical question. Finally, the yeast one-hybrid assay revealed that *BsMYB36* and *BsMYB51* could bind directly to the promoter region of *BsDFR4*, suggesting they might affect anthocyanin accumulation by regulating the expression of *BsDFR4.* However, the specific binding sites and regulatory mechanisms still require further investigation. To summarize comprehensively, these findings had significant implications for understanding the underlying causes of flower color and the pattern of differentiation in *B. striata*, provided actionable guidance for optimizing standardized cultivation parameters, and offered critical theoretical support for advancing *B. striata* germplasm resource research and molecular breeding strategies.

## Figures and Tables

**Table 1:** Statistics of SNP and InDel variation results in *B. striata* with different flowers.

SNP			InDel		
Category	P	WP	Category	P	WP
Upstream	198,672	250,086	Upstream	43,577	47,785
Exonic stop gain	1419	1820	Exonic stop gain	121	112
Exonic stop loss	317	406	Exonic stop loss	33	41
Exonic synonymous	61,507	85,170	Exonic frameshift deletion	2346	2615
Exonic nonsynonymous	65,284	87,614	Exonic frameshift insertion	2379	2478
Exonic unknowns	1935	2555	Exonic non-frameshift deletion	1468	1976
Intronic	3,137,853	4,039,573	Exonic non-frameshift insertion	987	1237
Splicing	591	785	Intronic	494,140	562,821
Downstream	188,222	243,441	Splicing	217	266
Upstream/downstream	5802	7305	Downstream	44,229	50,248
Intergenic	14,395,373	18,238,305	Upstream/downstream	1340	1554
Others	37,127	50,889	Intergenic	1,561,077	1,705,642
ts	13,683,683	17,376,725	Others	11,821	14,150
tv	4,410,419	5,631,223	Insertion	1,073,936	1,174,588
ts/tv	3.103	3.086	Deletion	1,088,148	1,214,096
Heterozygous rate (‰)	4.332	7.931	Heterozygous rate (‰)	0.293	0.506
Total	18,094,102	23,007,948	Total	2,163,644	2,390,821

**Table 2:** Statistics of share and unique SNP variations in exon region of significantly high expression gene in *B. striata* flowers.

Enzyme	Gene ID	Synonymous SNV			Nonsynonymous SNV			Exonic Stop Gain		
		Share	P	WP	Share	P	WP	Share	P	WP
PAL	evm.model.CTG1782.2	18	12	14	12	6	5	0	0	1
4CL	evm.model.CTG1092.18	1	1	1	1	0	5	0	0	0
	evm.model.CTG1903.4	5	2	8	5	1	4	0	0	1
	evm.model.CTG1332.28	2	3	5	2	1	1	0	0	0
CYP73A	evm.model.CTG1552.5	2	3	5	0	1	1	0	0	0
	evm.model.CTG1116.4	2	0	1	1	0	1	0	0	0
CHS	evm.model.CTG2075.7	5	2	5	1	0	1	0	0	0
	evm.model.CTG955.14	3	2	0	0	2	0	0	0	0
	evm.model.CTG955.10	0	0	0	0	0	1	0	0	0
CHI	evm.model.CTG4395.1	1	0	1	0	0	1	0	0	0
F3′5′H	evm.model.CTG521.30	3	2	1	1	0	3	0	0	0
	evm.model.CTG974.2	3	0	2	3	6	5	1	0	0
DFR	evm.model.CTG159.9	0	3	2	0	0	2	0	0	1
ANS	evm.model.CTG849.20	2	1	3	2	1	2	0	0	0
BZ1	evm.model.CTG213.4	2	0	0	1	0	0	0	0	0
	evm.model.CTG213.3	2	0	0	1	0	0	0	0	0

## Data Availability

The data underlying this article will be shared upon reasonable request to the corresponding authors.

## Supplementary Materials

The following supporting information can be downloaded at https://www.mdpi.com/article/10.3390/ijms26083555/s1.
