# Supplementary material for: Resequencing and Functional Analysis Revealed That BsDFR4 Could Cause the Formation of Different Flower Colors in Bletilla striata (Orchidaceae)
Source: Int J Mol Sci. 2025 Apr 10;26(8):3555. doi: 10.3390/ijms26083555 (PMC12026481; doi:10.3390/ijms26083555)
Supplement: Supplementary file 1 [file ijms-26-03555-s001.zip › ijms-3493643-supplementary.pdf]

Table S1 The quality of the sequencing raw data.

Category	P	WP
Raw bases (bp)	70,043,542,800	67,280,738,700
Clean bases (bp)	69,597,590,100	66,778,860,900
Effective rate (%)	99.32	99.27
Error rate (%)	0.03	0.03
Q20 (%)	97.29	96.95
Q30 (%)	92.78	91.57
GC content (%)	36.58	35.99

Table S2 Statistical results of comparison between sequencing results and reference genomes.

Sample	P	WP
Mapped reads	451,372,177	420,288,367
Total reads	463,983,934	445,192,406
Mapping rate (%)	97.28	94.41
Average depth(X)	26.40	22.71
Coverage at least 1X (%)	86.95	88.85
Coverage at least 4X (%)	83.62	85.71

Table S3 Statistics of SV and CNV variation results in *B. striata* with different flower.

SV			CNV		
Category	P	WP	Category	P	WP
Upstream	1003	1226	Upstream	1170	897
Exonic	10562	9218	Exonic	3614	4078
Downstream	984	1129	Intronic	9933	7311
Intronic	8716	10020	Downstream	870	636
Upstream/Downstream	28	30	Upstream/Downstream	24	15
Intergenic	36233	41699	Intergenic	58923	48269
Splicing	2	5	Others	138	109
Others	140	173	Duplication number	7263	11295
INS	77	8	Deletion number	67409	50020
DEL	49246	55997	Duplication length(bp)	82324000	214437900
INV	8345	7495	Deletion length(bp)	495156000	419426400
ITX	23064	22264			
CTX	19655	20155			
Total	100387	105919	Total	74672	61315

Table S4 Statistics on the number of anthocyanin synthesis pathway enzyme genes in different species.

	Bstr	Ashe	Doff	Dcat	Pequ	Osat	Atha	Total
PAL	4	5	2	2	2	8	4	27
4CL	17	15	20	15	14	16	15	112
CYP73A	2	2	2	2	2	3	1	14
CHS	11	7	7	8	5	2	1	41
CHI	4	1	6	4	3	5	6	29
F3H	1	1	1	1	1	1	1	7
F3'H	7	4	8	6	3	2	1	31
F3'5'H	4	1	0	1	1	1	0	8
DFR	6	1	5	4	2	4	3	25
ANS	1	0	1	1	1	2	1	7
BZ1	4	2	4	3	4	10	22	49
Total	61	39	56	47	38	54	55	

Table S5 Statistics on the variation of significantly high expression genes in *B. striata* flowers.

Enzyme	Gene ID	SNP		Indel		SV		CNV	
		P	WP	P	WP	P	WP	P	WP
PAL	evm.model.CTG1782.2	48	50	0	0	60	57	1	1
4CL	evm.model.CTG1092.18	3	8	1	1	218	153	0	1
	evm.model.CTG1903.4	13	23	1	1	91	75	0	0
	evm.model.CTG1332.28	8	10	0	0	96	76	1	1
CYP73A	evm.model.CTG1552.5	6	8	0	0	52	42	0	0
	evm.model.CTG1116.4	3	5	0	0	228	159	0	0
CHS	evm.model.CTG2075.7	8	12	0	0	367	280	0	0
	evm.model.CTG955.14	7	3	0	0	128	95	1	1
	evm.model.CTG955.10	0	1	0	0	130	95	1	0
CHI	evm.model.CTG4395.1	1	3	0	1	0	0	0	0
F3'5'H	evm.model.CTG521.30	6	8	0	0	201	138	0	0
	evm.model.CTG974.2	13	14	1	2	178	142	1	0
DFR	evm.model.CTG159.9	3	4	0	0	138	93	0	0
ANS	evm.model.CTG849.20	6	9	0	0	188	144	0	0
BZ1	evm.model.CTG213.4	3	3	0	0	225	177	0	0
	evm.model.CTG213.3	3	3	1	1	225	177	0	0

Table S6 Statistics of SNP variations in exon region of significantly high expression gene in *B. striata* flowers.

Enzyme	Gene ID	P			WP		
		Synonymous SNV	Nonsynonymous SNV	Exonic Stop gain	Synonymous SNV	Nonsynonymous SNV	Exonic Stop gain
PAL	evm.model.CTG1782.2	30	18	0	32	17	1
4CL	evm.model.CTG1092.18	2	1	0	2	6	0
	evm.model.CTG1903.4	7	6	0	13	9	1
	evm.model.CTG1332.28	5	3	0	7	3	0
CYP73A	evm.model.CTG1552.5	5	1	0	7	1	0
	evm.model.CTG1116.4	2	1	0	3	2	0
CHS	evm.model.CTG2075.7	7	1	0	10	2	0
	evm.model.CTG955.14	5	2	0	3	0	0
	evm.model.CTG955.10	0	0	0	0	1	0
CHI	evm.model.CTG4395.1	1	0	0	2	1	0
F3'5'H	evm.model.CTG521.30	5	1	0	4	4	0
	evm.model.CTG974.2	3	9	1	5	8	1

DFR	evm.model.CTG159.9	3	0	0	2	2	1
ANS	evm.model.CTG849.20	3	3	0	5	4	0
BZ1	evm.model.CTG213.4	2	1	0	2	1	0
	evm.model.CTG213.3	2	1	0	2	1	0

Table S7 Primers for qRT-PCR.

Gene	Primers name	Primers sequences（5’→3’）
BsActin	*BsActin1*-F	GCTTGCTTATGTGGCCCTGGA
	BsActin1-R	CCTCTCGGCGCCAATTGTGA
BsDRF	*BsDRF*-F	TGGCTACGTGGGTTCATGGC
	BsDRF-R	GCGCGGGAGATCCAGCAAT
PhActin	*Actin1*-F	GTTCTTTCCCTATATGCTAGTGGC
	Actin1-R	GAAGGATGGCATGAGGAAGTG
PhCHS	*PhCHS*-F	ATCGGACTCACCTTCCAC
	PhCHS-R	AGCACATTTCTACTCGCG
PhF3’H	*PhF3’H*-F	ACCTTCCGCCTACATCCC
	PhF3’H-R	GAACTCCAACGGCCCATC
PhDFR	*PhDFR*-F	GACCCTGAGAACGAAGTGATAC
	PhDFR-R	TGGTGTTCCTCCACGTTTAC
BsDFR4	*proBsDFR4*-F	GTCGACAACATCATTCTCATAAATA
	proBsDFR4-R	TTCTTCTTTCTCTCTCCTCTACTTC

Table S8 The sequence of *BsDFR4* promoter region.

	**Sequences**
proBsDFR4	GTCGACAACATCATTCTCATAAATATTGATAACAGCCTACTTCAACA<br>AGTTACTAATAAGCTTGCACTGTAATTCTCTATCAAAGATCTTGGAATGC<br>TCTCATATTTATTGGAGTTGAAGTCATTACCTCCCCTCAAGGTCTTTTCTC<br>TCTCAACACAAATATATCCAATACCTTTTAGATAAAACTTATATGAGCAAT<br>ATAAATGAAGTCACCACTCCTATGTCTCCTTATTATATGCCTACCAACCTT<br>GATGGCATTGCTCTCATCGACCTAACTGAATAATGCTCCATAGTTGGTGG<br>AGTTCAGTAACACTCTTTCATCGGATCGAATATTCCTTTGTGATAAACAA<br>ACTCTCTCAATTTATGTATGCATCGACCTACCACTACACACTGGAGTGTT<br>CTCAAACATATTCTTCTCTACCTTCAAGGCACCATCAATCATGGATTAATA<br>ATTTGTTGCTACTCCACATTATCTCTACATGGATTCACTAATGCTGAATGG<br>GCAGGCAACATCAATGACCGCACCTCTACATATGCCATTTTTCTTAGCCA<br>CAATGCTATCTCATGGAGCTCTAAGAAACAATGCATGGTTGCCCGCTCC<br>ACCATATATTTCAATTGACTTTCATTTTATACGAGAAAAAGTTCAAAGTG<br>GAGATCTTTGTGTCTCTCATGTCTCCTCTAATAATCAACTTGCGGATGCA<br>CTCACCAAACTTCTCTCGATCGTTGCCTCCTCGGTCAACTTCATTTCAAG<br>ATTGATGTCTTCTCCCGAGATTTCATCTTTAAGGGGCATATTAGAGGAATA<br>TCATAAATTATATTCATTGTATTTTTTACTTACCTTTTTAGTCTTACCACATT<br>AAGTTGCCATTAAATTCAAAGTGTTAATATACCTTCTTTTCTTACACATTT<br>TGTATATATAAATATTATACAAACCCTCTAATGCAAACAGACTTTTCTTCT<br>TCCTCAATTATTTTCTCTTACATAAAATAGCCTAATTTCATCCTATAATAAT<br>GTAATTTGTTCTTAGTGAGTGAAACCTAGCCAGTGATTCTAATCTGATCA<br>TTGGACCTAATTAAAAGAGCCAACTTCCACGCTCCCAGACCAACCACCA<br>TTCCCCATCCGATGGTCAACAATTCAAGCAGAGCTAAATGATCCATACAA<br>TTCGTCATTTAAAAGTTCTACTTCCAAAACTTTTGTTATGTAAGCCGCAT<br>GTTACAAGCTTCAACAAACAAAGATCACGTGCAGACCGGAAGTTCACC<br>CAACAAAACCCACAAAAAATATCCACGTGTCAATGATTCCCTGGTTTCA<br>TTCATTGGTCGGACTTAAGGTTCTTATAGTATTTTCAGTGGCTCCATCTTC<br>ACTGTTGAGTGGCCTTAAGTTTACCTCCTAGTGAGCATAACCGCTAGTAG<br>AAATTTACATGCTTTGTATAAATTGGTAGCATCGTTGTGCTTAAAATTGGA<br>GTGAGCGAGCTGAAACTGAAGTAGAGGAGAGAGAAAGAAGAA

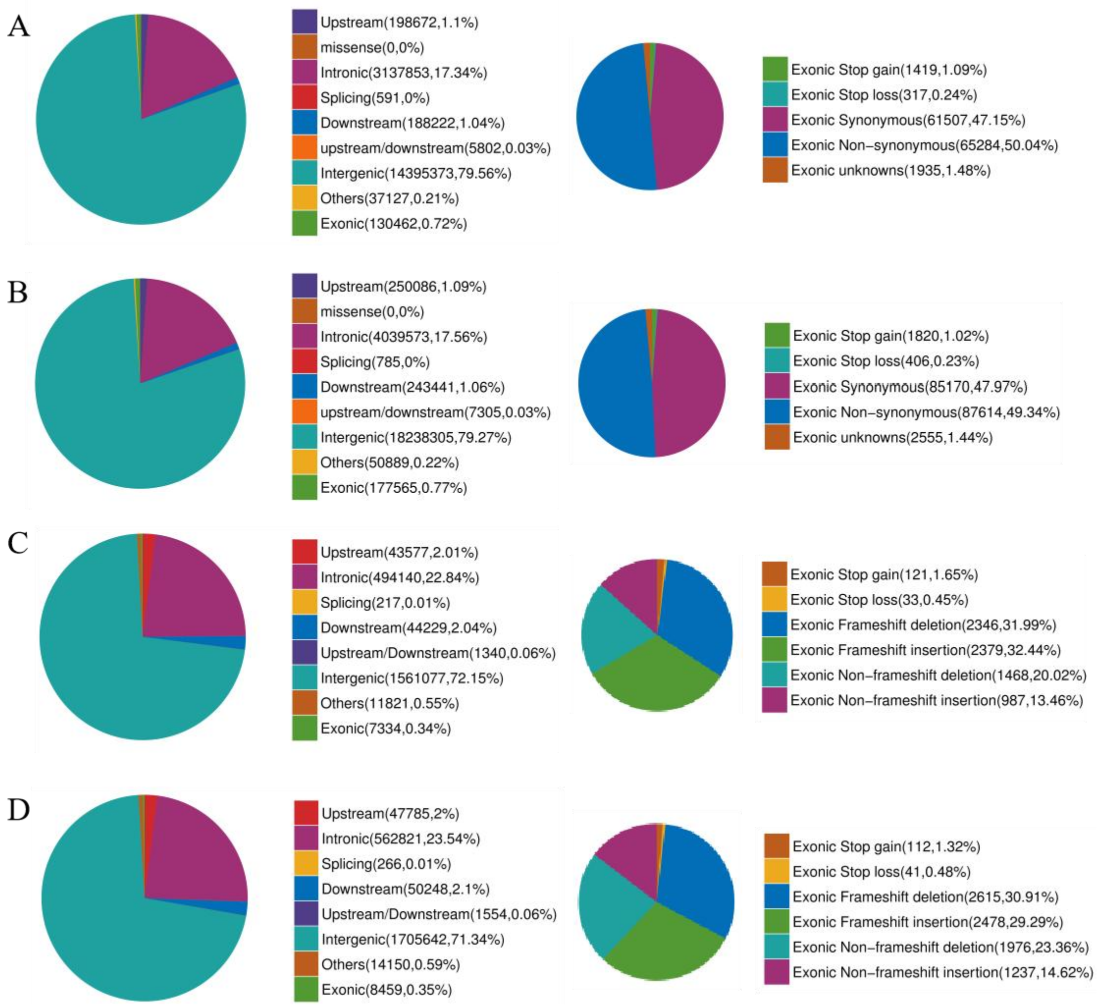

Figure S1 SNP and Indel variations in *B. striata* with different flower. The number of SNPs on different regions of the genome (left) and the number of different types of SNPs on coding regions (right) in group P (A) and group WP (B); The number of SNPs on different regions of the genome (left) and the number of different types of SNPs on coding regions (right) in group P (C) and group WP (D).

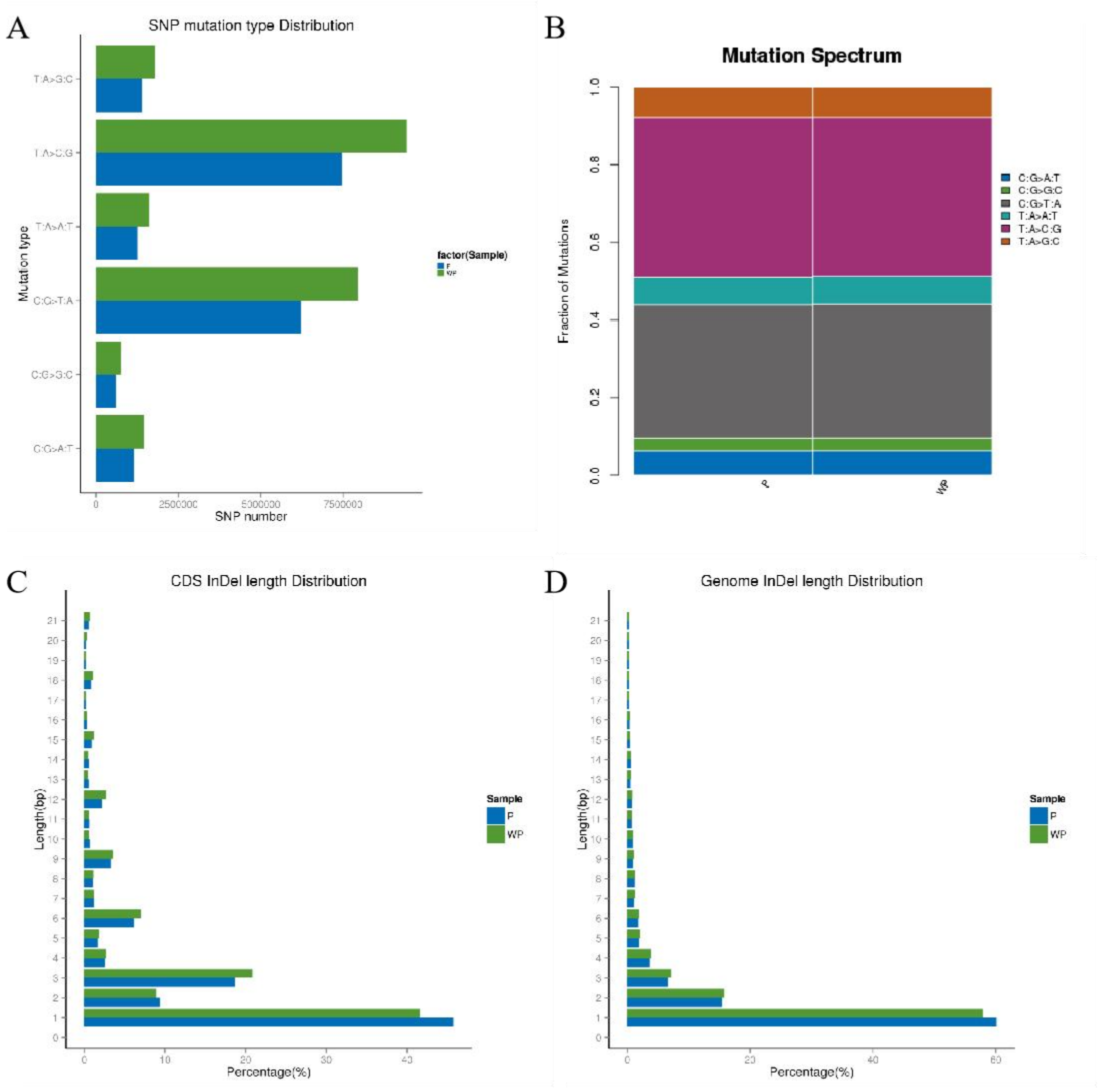

Figure S2 SNP mutation spectrum (A and B) and InDel length distribution (C and D).

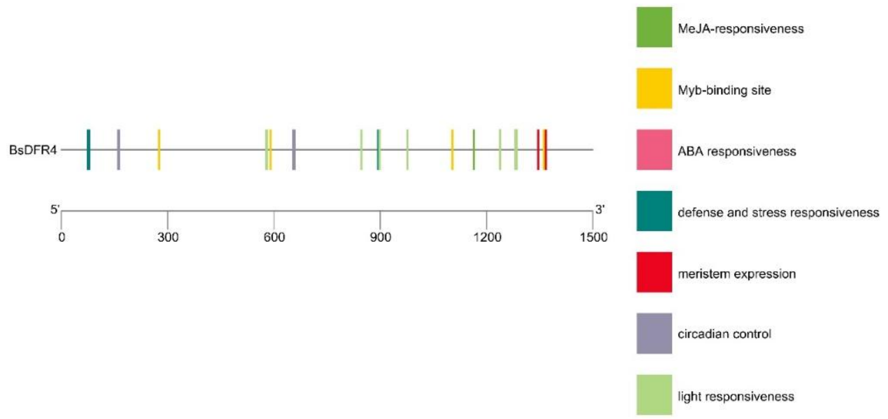

Figure S3 *Cis*-acting elements of *proBsDFR4*. The different colored boxes represent various response elements.
